# Long-Term Survival After Surgical Resection for Rectal Cancer Is Associated With Textbook Outcome but Not Surgical Case Volume

**DOI:** 10.1097/AS9.0000000000000601

**Published:** 2025-08-18

**Authors:** Mohamed Aly, Yu-Hui Chang, Chee-Chee Stucky, Zhi Ven Fong, David Etzioni, Nabil Wasif

**Affiliations:** From the * Department of Surgery, Mayo Clinic Arizona, Phoenix, AZ; † Department of Quantitative Health Sciences, Mayo Clinic Arizona, Scottsdale, AZ.

**Keywords:** long-term survival, rectal cancer, textbook outcome, volume

## Abstract

**Objective:**

Analyzing textbook outcome (TO) following rectal cancer resection and its association with long-term survival when compared to hospital case volume alone.

**Background:**

TO is a quality metric representing the ideal result following complex cancer surgery. Prior studies have suggested improved long-term survival for rectal cancer patients undergoing surgery at HV institutions.

**Methods:**

Patients undergoing surgery for rectal adenocarcinoma from 2014 to 2015 were identified using the National Cancer Database. Low (LV), medium (MV), and high-volume (HV) hospital strata were defined by quartile cutoffs (low <25th, high >75th, and 25–75th medium volume). TO was achieved with adequate lymph node count (≥12), negative margins (R0 resection), length of stay <75th percentile, absence of 30-day readmission/mortality event, and appropriate plus timely systemic therapy. Adjusted analyses for long-term survival were performed using a hierarchical multivariable Cox regression model.

**Results:**

TO was achieved in 28.5% of 48,484 patients. LV or MV hospital patients were more likely to be older, uninsured/Medicaid, and less likely to achieve a TO (HV 31.2% vs MV 29.6% vs LV 23.2%, *P* < 0.001). TO was associated with improved 5-year survival (84.0% vs 72.0%, *P* < 0.001). On multivariable analyses, TO was the strongest protective factor against mortality (HR 0.60, 95% confidence interval = 0.56–0.64), even after controlling for case volume.

**Conclusions:**

Only 28.5% of patients undergoing resection for rectal cancer achieve TO. However, they had a 40% reduction in long-term mortality independent of hospital volume. Optimizing long-term survival in patients with rectal cancer can be achieved by TO criteria rather than increasing surgical case volume.

## INTRODUCTION

Rectal cancer is diagnosed in approximately 44,000 patients annually in the United States and accounts for almost 14,000 deaths.^1,2^ There has been an increasing trend in the incidence of rectal cancer over the past decades, with a 20% increase over a period of 16 years. This is faster than the 2% annual increase also seen in the incidence of colon cancer, particularly in younger patients.^3,4^ Encouragingly, a reduction in 30-day and 90-day mortality after rectal cancer resection has also been reported and currently stands as 1.1% and 2.2%, respectively. In addition, after appropriate management including surgical resection and neoadjuvant therapy, there has been an increase in median survival following rectal cancer surgery of up to 92.1 months.^3^

As a result of these improvements in survival, research has been performed to identify the variables responsible for improved outcomes. Associations linking multimodality management and centers with higher case volumes to more favorable outcomes have been demonstrated.^5–7^ The association between higher case volume and improved postoperative mortality is the basis for recommendations made by bodies such as the Leapfrog group to limit rectal cancer surgery to high volume centers, defined as those with an annual case volume of 16 or more cases.^8,9^

Traditionally, morbidity and mortality rates have been used to quantify surgical outcomes following surgery. More recently, composite measures such as achieving a textbook outcome (TO) have been proposed as a more comprehensive and standardized metric to assess surgical outcomes. Not only does TO capture immediate postoperative outcomes, it can also incorporate variables that are associated with long-term survival after cancer surgery, hence better reflecting the totality of cancer care. This has been studied previously in cancers such as esophageal, gastric, and pancreatic cancer.^10–12^

Similar to the association of higher surgical case volume with improved operative mortality, there have also been studies showing an association with long-term survival with increasing case volume.^13^ However, higher surgical volume by itself has no biological rationale in improving long-term survival, and it is likely that the associations demonstrated previously are being mediated by intermediary variables. In this study, we hypothesize that a comprehensive outcome such as TO following rectal cancer resection results in a stronger association with long-term survival when compared to hospital case volume alone.

## METHODS

### Patient Selection

The National Cancer Database (NCDB) was used for this study, which is a cancer registry with outcomes data from more than 1500 Commission on Cancer (CoC) accredited facilities sponsored by the American College of Surgeons and the American Cancer Society. We extracted data on patients undergoing curative surgery for rectal adenocarcinoma from 2014 to 2015, to ensure a minimum of 5-year follow-up survival data. Only patients whose initial diagnosis and treatment were at the reporting facility were included. Patients were excluded if they had stage IV cancer, did not undergo curative intent surgery, or did not have valid 30-day mortality data. For each patient, we collected information on demographics, oncologic outcomes, postoperative outcomes, and long-term survival.

### Textbook Outcome

The requirements for TO were as follows: adequate lymph node count (12 or more nodes), negative margins (defined as microscopically negative or R0 resection), length of stay less than the 75th percentile (8 days), no 30-day readmission, no 30-day mortality, and appropriate and timely adjuvant therapy.^14^ Appropriate therapy was defined as receipt of either neoadjuvant or adjuvant therapy when indicated. For patients with clinical AJCC TNM T3/T4 or N+, preoperative chemoradiation therapy is indicated. For patients not undergoing neoadjuvant chemoradiation who are pathologic stage III, adjuvant chemotherapy is indicated. Timely therapy was defined as commencement of neoadjuvant therapy within 60 days of diagnosis or adjuvant therapy within 60 days of surgery. Patients with missing data for any of the components were considered as not having achieved TO. This approach may have underestimated the percentage of patients with a TO; thus, a sensitivity analysis was performed by excluding all patients with missing data for TO components (Supplemental Table 1, https://links.lww.com/AOSO/A522).

### Hospital Volume

Mean hospital volume was calculated according to the method proposed by Birkmeyer et al^8^ where mean annual volume for each hospital is calculated by dividing the total number of cases in a hospital by the years a hospital reports to the NCDB. The mean hospital volumes were ranked in ascending order, and the quartile values were identified by dividing the patients into 4 groups of equal size. Three volume groups were then determined: low (LV: <25th percentile), medium (MV: 25th – <75th percentile), and high (HV: 75th percentile).^15^

### Statistical Methods

Descriptive statistics were used to summarize patient demographic and clinical characteristics. For group comparisons, χ^2^ test was used for categorical variables. Overall survival was the outcome of interest and was defined by the time from diagnosis to the date of death or last contact. We used 5-year overall survival as our long-term survival endpoint. Patients who died within 30 days from surgery were excluded from all survival analyses. Kaplan-Meier curves for long-term survival were stratified by TO achievement and by hospital volume (low, medium, and high), and group comparisons were performed by the log-rank test. Two Cox proportional hazards models were fitted: 1 with and 1 without TO. The other covariates in the models included age, sex, race, insurance status, Charlson comorbidity score, pathology stage, and surgical volume. Type of surgery (ie, open vs minimally invasive) was also chosen as a variable to account for confounding by intention regarding surgical approach. Since the outcomes of patients treated in the same facility may be correlated and the assumption of independence is violated, we used a multilevel approach to account for the correlation. More specifically, patient data are clustered by facility. This approach provided pooled model estimates (effect size) across facilities and robust estimates of standard error. For each model, the point estimates of hazard ratio and 95% confidence interval (CI) were reported. The performance of each model was quantified by the concordance index, with higher values indicating better ability to discriminate between outcomes. Patients with a 30-day mortality following surgery were also excluded from multivariable analyses. The analyses were conducted in SAS 9.4 (SAS Institute, Cary, NC) and R 4.1.0 (The R Foundation for Statistical Computing, Vienna, Austria). All tests were 2-sided, and a *P* < 0.05 was considered statistically significant.

## RESULTS

### Descriptive Analysis

A total of 1310 hospitals and 48,484 patients with rectal resections were included in the study. The specific breakdown is as follows: low-volume (818 hospitals, 11,989 patients), medium-volume (414 hospitals, 21,183 patients), and high-volume (78 hospitals, 12,312 patients). The majority of patients (82.0%) were older than 50 years old. Males represented 62.2% of the total population. Patients treated at low-volume and medium-volume hospitals were more likely to be older, and uninsured or on Medicaid, compared with patients treated at high-volume hospitals. (Supplemental Table 2, https://links.lww.com/AOSO/A522). The mean number of cases per year ranged from 1-6.8 for the low-volume hospital group, 6.9 to 22.2 for medium-volume, and 22.3 to 83.2 for high-volume.

### Textbook Outcome

**FIGURE 1.**
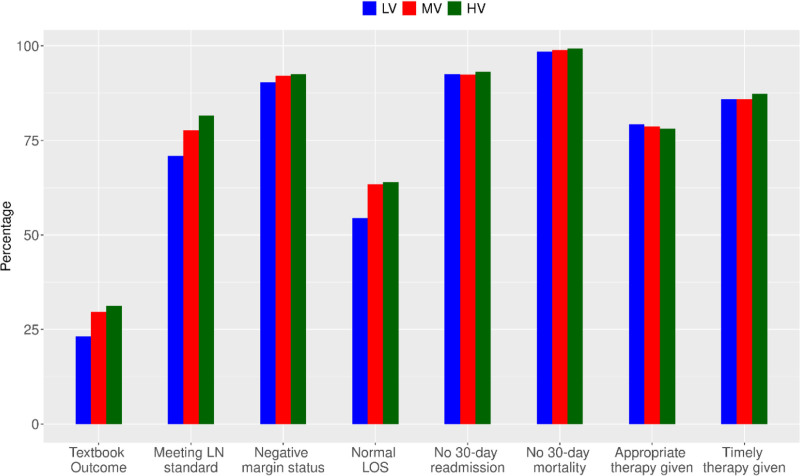
Distribution of textbook outcome by each individual outcome among hospital volume groups. HV indicates high-volume; LN, lymph node; LOS, length of stay; LV, low-volume; MV, medium-volume.

TO was achieved more often in patients undergoing surgery at higher volume hospitals (31.2% in high-volume vs 29.6% in medium-volume, and 23.2% in low-volume *P* < 0.001 for overall and pairwise comparisons). Other individual components of TO were also significantly better in higher volume versus medium volume versus low volume hospitals, with the largest discrepancy being in the rate of adequate lymph node retrieval (71% in low volume hospitals vs 82% in high volume hospitals). Supplemental Table 2, https://links.lww.com/AOSO/A522 shows the numerical data, and Figure 1 the graphical representation of these differences.

### Survival Analysis

**FIGURE 2.**
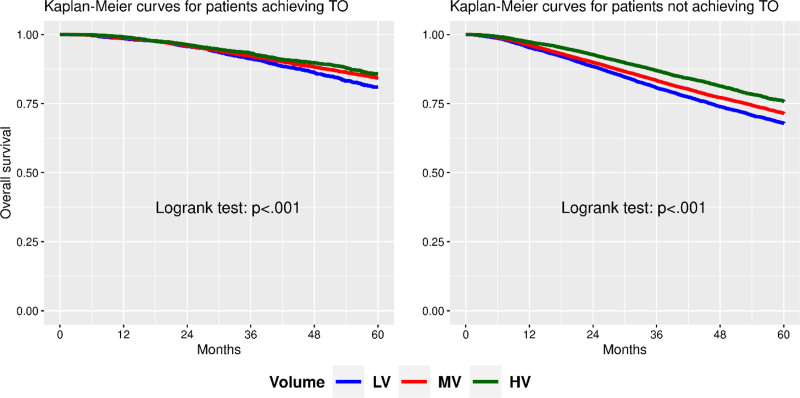
Kaplan-Meier survival curves stratified by hospital procedure volume in both groups, patients achieving TO and patients not achieving TO.

**FIGURE 3.**
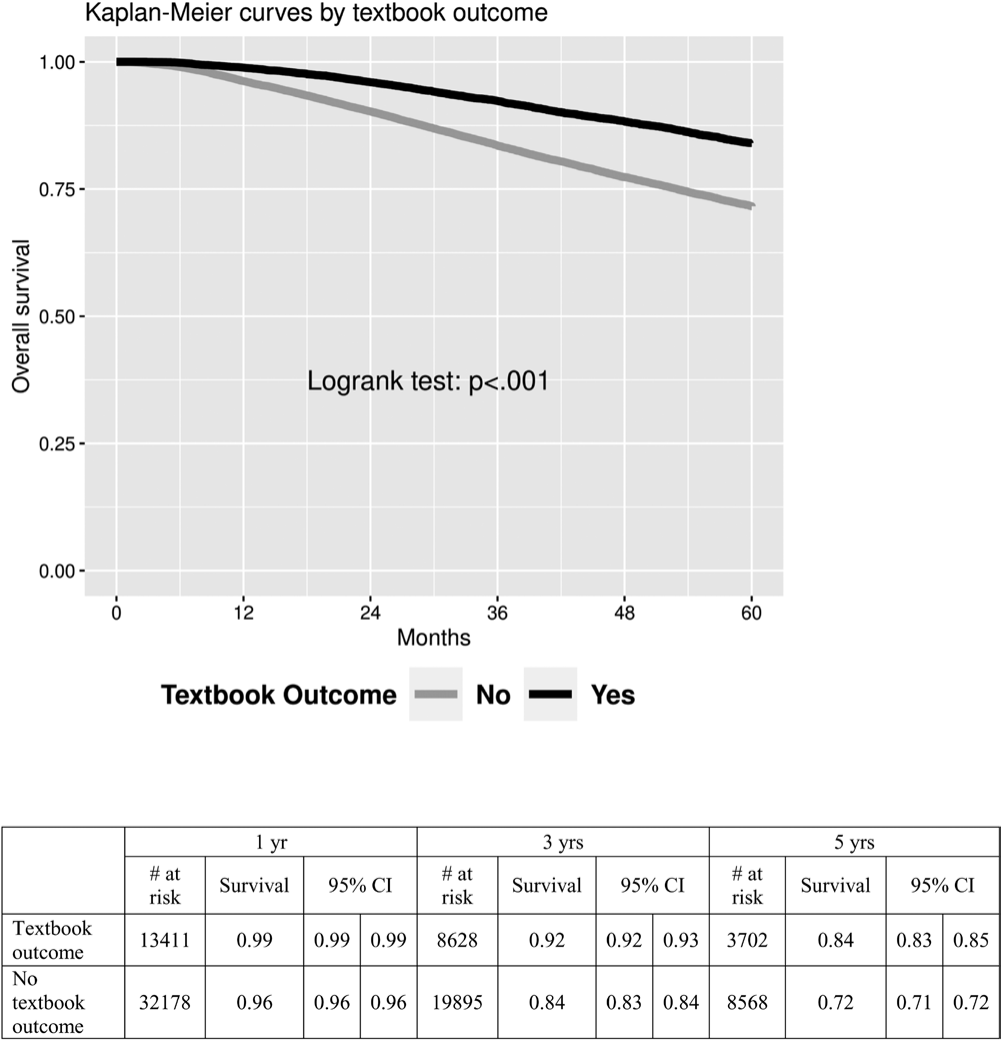
Kaplan-Meier survival curves stratified by textbook outcome.

Patients who achieved TO had improved overall survival at 5 years, with a 12% advantage over those who did not (84% vs 72%, *P* < 0.001). When stratified by hospital volume alone, patients at high-volume centers had increased survival at 3 and 5 years compared with low-volume and medium-volume hospitals (Figs. 2 and 3).

### Multivariable Cox Regression Analysis

An adverse association with long-term survival after rectal cancer surgery was seen for patients who were older, male, underinsured, had increased comorbidity, underwent open surgery, and with increasing pathologic stage (Table 1). When controlling for both hospital volume and TO, achieving TO was associated with a 40% reduction in the risk of mortality at 5 years (HR 0.60; 95% CI = 0.56–0.64), and the performance of the model was slightly better with a concordance of 0.72 (95% CI = 0.714–0.727). To further investigate this, an interaction term was introduced in the model of TO and hospital volume. This showed that in hospitals achieving TO, hospital volume was not associated with improved survival. It was only in hospitals that did not achieve TO that there was an association between hospital volume and improved survival (Table 2).

**TABLE 1.** Hierarchical Cox Regression Model for Overall Survival Without TO (Model A) and With TO (Model B)*

Variable	Comparison	Model A				Model B			
		HR	95% CI		*P*	HR	95% CI		*P*
Age	Per 1 year older	1.04	1.03	1.04	<0.001	1.04	1.03	1.04	<0.001
Sex	Female versus male	0.78	0.74	0.82	<0.001	0.79	0.75	0.83	<0.001
Race	Black versus White	1.28	1.17	1.39	<0.001	1.23	1.14	1.34	<0.001
	Others versus White	0.75	0.66	0.85	<0.001	0.74	0.66	0.84	<0.001
Insurance	Not insured/Medicaid versus private/managed care	1.47	1.34	1.61	<0.001	1.41	1.29	1.55	<0.001
	Medicare/other government versus private/managed care	1.37	1.28	1.47	<0.001	1.35	1.26	1.44	<0.001
Charlson-deyo comorbidity	1 versus 0	1.20	1.13	1.28	<0.001	1.19	1.12	1.27	<0.001
	2 versus 0	1.33	1.21	1.47	<0.001	1.31	1.19	1.45	<0.001
	3+ versus 0	1.94	1.72	2.18	<0.001	1.89	1.68	2.12	<0.001
Surgery	MIS versus open	0.78	0.74	0.83	<0.001	0.81	0.77	0.86	<0.001
Pathology stage	2 versus 0/1	1.78	1.64	1.94	<0.001	1.73	1.59	1.87	<0.001
	3 versus 0/1	2.80	2.60	3.03	<0.001	2.79	2.58	3.01	<0.001
	Unknown versus 0/1	1.22	1.10	1.35	<0.001	1.20	1.08	1.33	<0.001
Volume	MV versus LV	0.88	0.82	0.94	<0.001	0.90	0.84	0.96	<0.001
	HV versus LV	0.76	0.69	0.84	<0.001	0.78	0.71	0.85	<0.001
Textbook outcome	Yes versus No					0.60	0.56	0.64	<0.001
Concordance (95% CI)		0.713 (0.706–0.720)				0.720 (0.714–0.727)			

* Patients who died within 30 days were excluded in the multivariable Cox model.

**TABLE 2.** Hierarchical Cox Regression Model for Overall Survival With an Interaction Term for TO Versus Hospital Volume (Model C)

Variable	Comparison	Model C			
		HR	95% CI		*P*
Age	Per 1 year older	1.04	1.03	1.04	<0.001
Sex	Female versus male	0.79	0.75	0.83	<0.001
Race	Black versus White	1.24	1.14	1.34	<0.001
	Others versus White	0.74	0.67	0.83	<0.001
Insurance	Not insured/Medicaid versus private/managed care	1.41	1.30	1.54	<0.001
	Medicare/other government versus private/managed care	1.35	1.26	1.44	<0.001
Charlson-Deyo comorbidity	1 versus 0	1.19	1.12	1.27	<0.001
	2 versus 0	1.31	1.19	1.44	<0.001
	3+ versus 0	1.89	1.71	2.10	<0.001
Surgery	MIS versus Open	0.82	0.78	0.86	<0.001
Pathology stage	2 versus 0/1	1.73	1.59	1.87	<0.001
	3 versus 0/1	2.79	2.58	3.01	<0.001
	Unknown versus 0/1	1.20	1.08	1.33	<0.001
Textbook outcome = Yes					
Volume	MV versus LV	1.00	0.86	1.17	0.95
	HV versus LV	0.98	0.83	1.16	0.83
Textbook outcome = No					
Volume	MV versus LV	0.88	0.83	0.94	<0.001
	HV versus LV	0.74	0.69	0.80	<0.001
Concordance (95% CI)		0.721 (0.714–0.728)			

## DISCUSSION

In this study, we show that a composite outcome such as TO displays a robust association with long-term survival independent of hospital volume after rectal cancer resection. Although high-volume centers achieve TO at a significantly higher rate than the lower volume hospitals, TO achievement was associated with a 40% reduction in the chance of mortality in all hospitals even after controlling for volume. In hospitals that did achieve TO, volume was not associated with improved long-term survival. It was only in hospitals not achieving TO that a higher volume of cases was associated with improved long-term survival. This suggests that efforts directed towards improving TO will improve long-term survival for patients with rectal cancer overall, independent of hospital case volume.

Prior work using the Medicare database has demonstrated an association between long-term survival and high surgical case volume of pancreatectomies and hepatectomies.^13^ Similarly, a study by our group of 6432 patients from the SEER-Medicare database who underwent operative treatment for rectal cancer showed improved long-term survival at high volume centers (defined as ≥25 cases/year).^7^ However, in this study, we show that the strongest predictor of long-term survival in patients with rectal cancer undergoing surgery is TO and this association persists even when controlling for hospital volume. One approach to interpret these findings is to consider TO as a mediator or intermediary variable in the association between hospital volume and improved long-term survival. As seen in Figure 1, high-volume hospitals were more likely to achieve individual TO components in comparison to low and medium-volume hospitals. Once we introduced an interaction term in our model, hospitals that achieved a TO did not show any association between volume and improved survival. This suggests that there are processes and measures in place at high-volume hospitals, such as appropriate patient selection for chemotherapy and timely receipt of therapy, that correlate with long-term survival in rectal cancer. Other components of the TO, such as R0 resection margins and adequate nodal counts, are well-known markers of cancer recurrence and worse survival, lending biological plausibility to our findings.

Our findings also have direct implications with regard to the regionalization of rectal cancer surgery. A policy measure focused solely on increasing surgical case volume is unlikely to improve long-term survival in this patient population. However, targeted interventions towards improving surgical technique (complete mesorectal resection for R0 margins), pathology protocols (to ensure adequate nodal harvest), and coordination of care in all hospitals to ensure timely and adequate receipt of systemic therapy are likely to yield a 12% survival benefit at 5 years for patients achieving a TO irrespective of case volume. Implementation of these quality improvement efforts across the board has the potential to improve outcomes at medium and low volume hospitals. This is particularly important in light of the fact that regionalization has been shown to have unintended consequences such as disparities in access, fragmented care, and an increased travel burden for patients. In addition, regionalization of rectal cancer surgery may not be applicable at times due to obstacles such as type of insurance coverage, distance traveled, and patient preference to stay local for support or financial limitations. On the other hand, the individual components of TO can be achieved in the absence of regionalization by focusing efforts on institutional processes and measures. For instance, adopting a standardized pathology protocol that is recommended by the College of American Pathologists can optimize nodal yield and margin analysis. Similarly, improvement in surgical technique can be achieved by subspeciality training. Coordination of care, rather than depending just on medical expertise, plays a crucial role for timely chemotherapy administration. Emphasizing such components facilitates achievable goals, which represent integral components of CoC accreditation, a standard hallmark among NCDB-reporting hospitals. Furthermore, work by our group and others has shown that even for short-term outcomes such as 30-day mortality, there is an inconsistent relationship between hospital case volume and surgical quality.^16–19^ Finally, the association between high volume hospitals and more frequent achievement of TO suggests that there are knock-on benefits of higher patient volumes, such as an improvement in processes and protocols that will lead to better long-term outcomes for patients (eg, better coordination of chemotherapy).

## LIMITATIONS

We acknowledge several limitations in our study. Although the NCDB has the advantage of providing large sample sizes, the data are subject to misclassification, selection bias, and reporting bias. Patient enrollment into the database is from CoC-accredited hospitals only, which may limit generalization of these findings. Our outcome measure is overall survival as disease-specific survival is not available in the dataset. Our choice for the individual components of TO was informed by clinical experience and is based on published studies on the topic to date. It is possible that the selection of different variables to define TO could change the point estimates. However, this is unlikely to change the overall message of the study. Also, it is possible that length of stay for patients could have been impacted by the complexity and extent of surgery (eg, pelvic exenteration) and patient factors (eg, morbid obesity). Due to a lack of granularity in the available dataset, we are unable to study this further.

## CONCLUSION

In conclusion, for individual patients, achieving TO after surgery for rectal cancer is associated with a 40% reduction in long-term mortality independent of the annual case volume of the treating hospital. This suggests that focusing quality improvement efforts towards individual metrics of TO would likely yield greater benefit than any push towards regionalization to high-volume centers for rectal cancer patients.

## Supplementary Material
