# Supplementary material for: Long-Term Survival After Surgical Resection for Rectal Cancer Is Associated With Textbook Outcome but Not Surgical Case Volume
Source: Ann Surg Open. 2025 Aug 18;6(3):e601. doi: 10.1097/AS9.0000000000000601 (PMC12453300; doi:10.1097/AS9.0000000000000601)
Supplement: Supplementary file 1 [file as9-6-e601-s001.pdf]

SUPPLEMENTAL TABLES:

Supplemental Table 1. Sensitivity analysis results after excluding 5878 patients with missing data in the variables constituting textbook outcome components.

Variable	Comparison	Model A (sensitivity analysis)				Model B (sensitivity analysis)			
		HR	95% CI		p-value	HR	95% CI		p-value
Age	Per 1 year older	1.04	1.04	1.04	<.001	1.04	1.03	1.04	<.001
Sex	Female vs. male	0.77	0.73	0.81	<.001	0.78	0.74	0.82	<.001
Race	Black vs. white	1.26	1.15	1.37	<.001	1.21	1.11	1.32	<.001
	Others vs. white	0.75	0.67	0.86	<.001	0.75	0.66	0.85	<.001
Insurance	Not insured/Medicaid vs. private/managed care	1.42	1.29	1.56	<.001	1.37	1.24	1.50	<.001
	Medicare/other government vs. private/managed care	1.34	1.25	1.44	<.001	1.32	1.23	1.41	<.001
Charlson-Deyo comorbidity	1 vs. 0	1.19	1.12	1.27	<.001	1.18	1.11	1.26	<.001
	2 vs. 0	1.33	1.20	1.47	<.001	1.31	1.18	1.44	<.001
	3+ vs. 0	1.91	1.69	2.16	<.001	1.86	1.65	2.10	<.001
Surgery	MIS vs. Open	0.79	0.75	0.83	<.001	0.82	0.78	0.87	<.001
Pathology stage	2 vs. 0/1	1.79	1.64	1.94	<.001	1.72	1.58	1.88	<.001
	3 vs. 0/1	2.84	2.63	3.08	<.001	2.83	2.61	3.07	<.001
	Unknown vs. 0/1	1.22	1.09	1.35	<.001	1.20	1.08	1.33	<.001
Volume	MV vs. LV	0.88	0.82	0.94	<.001	0.90	0.84	0.96	<.001
	HV vs. LV	0.77	0.70	0.84	<.001	0.78	0.71	0.86	<.001
Textbook outcome	Yes vs. No					0.60	0.56	0.64	<.001
**Concordance (95% CI)**		0.715 (0.708, 0.722)				0.722 (0.715, 0.729)			

Supplemental Table 2. Descriptive statistics for low, medium and high-volume hospital cohorts

	LV (N=11989)	MV (N=24183)	HV (N=12312)	Total (N=48484)	p-value
**Number of hospitals**	**818**	**414**	**78**	**1310**	
**Year of Diagnosis**, n (%)					0.001[1]
2014	2012 (16.8%)	3913 (16.2%)	1954 (15.9%)	7879 (16.3%)	
2015	1923 (16.0%)	3876 (16.0%)	1897 (15.4%)	7696 (15.9%)	
2016	1977 (16.5%)	3864 (16.0%)	2025 (16.4%)	7866 (16.2%)	
2017	1994 (16.6%)	3775 (15.6%)	2082 (16.9%)	7851 (16.2%)	
2018	2056 (17.1%)	4386 (18.1%)	2228 (18.1%)	8670 (17.9%)	
2019	2027 (16.9%)	4369 (18.1%)	2126 (17.3%)	8522 (17.6%)	
**Age**, n (%)					<.001[1]
<50	1806 (15.1%)	4242 (17.5%)	2680 (21.8%)	8728 (18.0%)	
50-64	5056 (42.2%)	10413 (43.1%)	5230 (42.5%)	20699 (42.7%)	
65-79	4041 (33.7%)	7608 (31.5%)	3582 (29.1%)	15231 (31.4%)	
80+	1086 (9.1%)	1920 (7.9%)	820 (6.7%)	3826 (7.9%)	
**Sex**, n (%)					0.40[1]
Male	7512 (62.7%)	14983 (62.0%)	7681 (62.4%)	30176 (62.2%)	
Female	4477 (37.3%)	9200 (38.0%)	4631 (37.6%)	18308 (37.8%)	
**Race**, n (%)					<.001[1]
White	10330 (86.2%)	20346 (84.1%)	10361 (84.2%)	41037 (84.6%)	
Black	969 (8.1%)	2040 (8.4%)	928 (7.5%)	3937 (8.1%)	
Others	690 (5.8%)	1797 (7.4%)	1023 (8.3%)	3510 (7.2%)	
**Insurance**, n (%)					<.001[1]
Not insured	350 (2.9%)	811 (3.4%)	303 (2.5%)	1464 (3.0%)	
Private/managed care	5175 (43.2%)	11673 (48.3%)	6489 (52.7%)	23337 (48.1%)	
Medicaid	1174 (9.8%)	2142 (8.9%)	993 (8.1%)	4309 (8.9%)	
Medicare/other government	5146 (42.9%)	9284 (38.4%)	4383 (35.6%)	18813 (38.8%)	
Missing	144 (1.2%)	273 (1.1%)	144 (1.2%)	561 (1.2%)	
**Charlson-Deyo Score**, n (%)					0.01[1]
0	9217 (76.9%)	18582 (76.8%)	9632 (78.2%)	37431 (77.2%)	

	LV<br>(N=11989)	MV<br>(N=24183)	HV<br>(N=12312)	Total<br>(N=48484)	p-value
1	1979 (16.5%)	3876 (16.0%)	1859 (15.1%)	7714 (15.9%)	
2	487 (4.1%)	1056 (4.4%)	484 (3.9%)	2027 (4.2%)	
≥3	306 (2.6%)	669 (2.8%)	337 (2.7%)	1312 (2.7%)	
**Median Income Quartiles 2016 - 2020**, n (%)					<.001[1]
< $46,277	1802 (15.0%)	3491 (14.4%)	1844 (15.0%)	7137 (14.7%)	
$46,227-$57,856	2658 (22.2%)	4472 (18.5%)	2318 (18.8%)	9448 (19.5%)	
$57,857-$74,062	2496 (20.8%)	4956 (20.5%)	2426 (19.7%)	9878 (20.4%)	
>= $74,063	3305 (27.6%)	7351 (30.4%)	3976 (32.3%)	14632 (30.2%)	
Missing	1728 (14.4%)	3913 (16.2%)	1748 (14.2%)	7389 (15.2%)	
**Percent No High School Degree 2016-2020**, n (%)					<.001[1]
≥ 15.3%	2236 (18.7%)	4559 (18.9%)	2312 (18.8%)	9107 (18.8%)	
9.1%-15.2%	3138 (26.2%)	5777 (23.9%)	2928 (23.8%)	11843 (24.4%)	
5.0%-9.0%	3084 (25.7%)	5811 (24.0%)	2931 (23.8%)	11826 (24.4%)	
< 5.0%	1836 (15.3%)	4184 (17.3%)	2428 (19.7%)	8448 (17.4%)	
Missing	1695 (14.1%)	3852 (15.9%)	1713 (13.9%)	7260 (15.0%)	
**Surgery**, n (%)					<.001[1]
MIS	3994 (33.3%)	12770 (52.8%)	6903 (56.1%)	23667 (48.8%)	
Open	3963 (33.1%)	8270 (34.2%)	4126 (33.5%)	16359 (33.7%)	
Missing	4032 (33.6%)	3143 (13.0%)	1283 (10.4%)	8458 (17.4%)	
**Analytic Pathology Stage, n (%)**					<.001[1]
0	220 (1.8%)	465 (1.9%)	247 (2.0%)	932 (1.9%)	
1	2058 (17.2%)	4423 (18.3%)	2345 (19.0%)	8826 (18.2%)	
2	3372 (28.1%)	6723 (27.8%)	3385 (27.5%)	13480 (27.8%)	
3	4288 (35.8%)	8539 (35.3%)	4376 (35.5%)	17203 (35.5%)	
Unknown	1900 (15.8%)	3690 (15.3%)	1848 (15.0%)	7438 (15.3%)	
Missing	151 (1.3%)	343 (1.4%)	111 (0.9%)	605 (1.2%)	
**Neoadjuvant chemotherapy**	8555 (71.4%)	17760 (73.4%)	9151 (74.3%)	35466 (73.1%)	<.001

	LV	MV	HV	Total	p-value
	(N=11989)	(N=24183)	(N=12312)	(N=48484)	
**5-year mortality**	2679 (22.3%)	4545 (18.8%)	1931 (15.7%)	9155 (18.9%)	<.001
**Textbook outcome**, n (%)					<.001[1]
No	9209 (76.8%)	17013 (70.4%)	8466 (68.8%)	34688 (71.5%)	
Yes	2780 (23.2%)	7170 (29.6%)	3846 (31.2%)	13796 (28.5%)	
**Meeting LN standard**, n (%)					<.001[1]
Below LN standard	3300 (27.5%)	5211 (21.5%)	2187 (17.8%)	10698 (22.1%)	
Meeting LN standard	8500 (70.9%)	18791 (77.7%)	10046 (81.6%)	37337 (77.0%)	
Unknown	189 (1.6%)	181 (0.7%)	79 (0.6%)	449 (0.9%)	
**Margin Status**, n (%)					<.001[1]
Negative	10839 (90.4%)	22272 (92.1%)	11393 (92.5%)	44504 (91.8%)	
Positive	913 (7.6%)	1637 (6.8%)	794 (6.4%)	3344 (6.9%)	
Unknown	237 (2.0%)	274 (1.1%)	125 (1.0%)	636 (1.3%)	
**Normal LOS (< Q3, 7 days)**, n (%)					<.001[1]
Prolonged LOS	3488 (29.1%)	6746 (27.9%)	3556 (28.9%)	13790 (28.4%)	
Normal LOS	6533 (54.5%)	15338 (63.4%)	7882 (64.0%)	29753 (61.4%)	
Missing	1968 (16.4%)	2099 (8.7%)	874 (7.1%)	4941 (10.2%)	
**30-day readmission**, n (%)					<.001[1]
No	11091 (92.5%)	22360 (92.5%)	11470 (93.2%)	44921 (92.7%)	
Yes	632 (5.3%)	1462 (6.0%)	705 (5.7%)	2799 (5.8%)	
Unknown	266 (2.2%)	361 (1.5%)	137 (1.1%)	764 (1.6%)	
**30-day mortality**, n (%)					<.001[1]
No	11811 (98.5%)	23925 (98.9%)	12222 (99.3%)	47958 (98.9%)	
Yes	178 (1.5%)	258 (1.1%)	90 (0.7%)	526 (1.1%)	
**Appropriateness of therapy**					0.09[1]
No	2491 (20.8%)	5150 (21.3%)	2701 (21.9%)	10342 (21.3%)	
Yes	9498 (79.2%)	19033 (78.7%)	9611 (78.1%)	38142 (78.7%)	

	LV<br>(N=11989)	MV<br>(N=24183)	HV<br>(N=12312)	Total<br>(N=48484)	p-value
**Timeliness of therapy**					<0.00[1]
No	1690 (14.1%)	3404 (14.1%)	1558 (12.7%)	6652 (13.7%)	
Yes	10299 (85.9%)	20779 (85.9%)	10754 (87.3%)	41832 (86.3%)	

MIS: Minimally Invasive Surgery
